# Zenker’s Diverticulum: Can Protocolised Measurements with Barium SWALLOW Predict Severity and Treatment Outcomes? The “Zen-Rad” Study

**DOI:** 10.1007/s00455-020-10148-5

**Published:** 2020-06-19

**Authors:** Sauid Ishaq, Keith Siau, Minhong Lee, Hamid M Shalmani, Toshio Kuwai, Lindsey Priestnall, Humayun Muhammad, Adrian Hall, Chris J Mulder, Helmut Neumann, Akhmid Aziz

**Affiliations:** 1 Gastroenterology Department, Russells Hall Hospital, Dudley, UK; 2 Birmingham City University, Birmingham, UK; 3 St George’s University, West Indies, Grenada; 4 Gastroenterology and Liver Diseases, Shahid Beheshti University of Medical Sciences, Tehran, Iran; 5 Department of Gastroenterology, National Hospital Organization, Kure Medical Center and Chugoku Cancer Center, Kure, Japan; 6 Radiology Department, Russells Hall Hospital, Dudley, UK; 7 Department of Health and Science, Roehampton University, London, UK; 8 Department of Gastroenterology and Hepatology, VU Medical Center, Amsterdam, The Netherlands; 9 Department of Interdisciplinary Endoscopy, University Hospital Mainz, Mainz, Germany

**Keywords:** Zenker diverticulum, Dysphagia, Myotomy, Deglutition, Deglutition disorder

## Abstract

Although barium swallow imaging is established in the investigation of Zenker’s diverticulum (ZD), no agreed measurement protocol exists. We developed a protocol for measuring ZD dimensions and aimed to correlate measurements with symptoms and post-operative outcomes. This prospective study included patients with confirmed ZD who underwent flexible endoscopic septal division (FESD) between 2014 and 2018. ZD was confirmed on barium radiology with measurements reviewed by two consultant radiologists. Symptom severity pre- and post-FESD was measured using the Dysphagia, Regurgitation, Complications (DRC) scale. Regression analyses were conducted to identify dimensions associated with therapeutic success, defined as remission (DRC score ≤ 1) 6 months after index FESD. In total, 67 patients (mean age 74.3) were included. Interobserver reliability (intraclass correlation coefficients—ICCs) was greatest for pouch width (0.981) and pouch depth (0.934), but not oesophageal depth (0.018). Male gender (60.9%) was associated with larger pouch height (*P* = 0.008) and width (*P* = 0.004). A positive correlation was identified between baseline DRC score and pouch depth (*ρ* 0.326, *P* = 0.011), particularly the regurgitation subset score (*ρ* 0.330, *P* = 0.020). The index pouch depth was associated with FESD procedure time (rho 0.358, *P* = 0.041). Therapeutic success was achieved in 64.2% and was associated with shorter pouch height (median 14.5 mm vs. 19.0 mm, *P* = 0.030), pouch width (median 19.9 mm vs. 28.8 mm, *P* = 0.34) and cricopharyngeal length (median 20.2 mm vs. 26.3 mm, *P* = 0.036). ZD dimensions may be feasible and were evaluated using Barium radiology. Specific parameters appear to correlate with severity and post-FESD outcomes, which aid with pre-procedural planning.

**Electronic supplementary material:**

The online version of this article (10.1007/s00455-020-10148-5) contains supplementary material, which is available to authorized users.

## Introduction

Zenker’s diverticulum (ZD) is the most common diverticulum of the upper gastrointestinal tract and is a treatable cause of dysphagia [1, 2]. Over the last two decades, the treatment paradigm for ZD has shifted away from surgical stapling towards less invasive endoscopic means [3]. With its favourable efficacy and safety profile [4], flexible endoscopic septal division (FESD) has established itself as the primary therapeutic modality for both treatment naïve and recurrent ZD [3, 5].

Currently, barium swallow remains the mainstay imaging modality for patients with suspected ZD. Multi-frame fluoroscopic imaging is typically performed with at least lateral and anteroposterior projections of the hypopharynx and cervical oesophagus, although oblique views may also be helpful to adequately image the cricopharyngeus muscle (CP). In addition to the initial diagnosis, this technique differentiates ZD seen on the posterior wall, with the neck of the diverticulum seen above the level of the cricopharyngeus muscle, from the less common, smaller and less symptomatic Killian-Jamieson diverticulum that arises from the lateral wall below the level of the CP. Following on from the diagnosis, the typical radiological barium swallow assessment of ZD usually involves anatomical and functional elements with the lateral projection being most useful. The anatomical assessment traditionally consists of an estimate of pouch size and neck width. Functional assessment includes Queryevaluating for pooling of contrast, regurgitation and aspiration, with exploration of the effects of the diverticulum on the adjacent oesophagus which may contribute to symptomatic dysphagia [6]. Specific assessment and measurement of the cricopharyngeus muscle that is central to FESD is less commonly undertaken.

Despite its role in the diagnosis and functional assessment of ZD, there is no accepted protocol for the standardised reporting of ZD dimensions based on barium swallow. This is relevant as certain dimensions correlate with treatment outcomes. The study by Costamagna et al. found “ZD size” to be an independent factor associated with FESD treatment failure [7]. It was assumed that the ZD size represented the width of the pouch, although the precise plane of measurement was not defined. There is, therefore, a need to establish a protocol to standardise the nomenclature of ZD dimensions, both for radiological and endoscopic usage, and to facilitate such measurements. Accordingly, this study aimed to develop a protocol for the fluoroscopic measurement of ZD dimensions, assess for interobserver reliability and to correlate these measurements with pre-treatment symptoms and post-treatment outcomes.

## Methods

### Study Design

This was a prospective single-centre observational study of patients with symptomatic ZD who underwent barium swallow and subsequent flexible endoscopic septal division (FESD) therapy. Patients were deemed symptomatic if they presented with dysphagia or regurgitation symptoms, with or without complications of aspiration or weight loss (DRC > 1). Patients with treatment naïve and recurrent ZD (post-surgical stapling) were included. FESD procedures were performed by a single operator within a tertiary referral centre (Dudley Group Hospitals NHS Foundation Trust, Dudley) between 2014 and 2018. The procedure was performed using the standardised FESD technique as previously described [4, 5]. ZD dimensions were derived from barium swallow examinations using the protocol below, with consensus from two consultant radiologists. All patients received propofol sedation with anaesthesiologist assistance, with the a priori intention of achieving symptom remission after index therapy. All patients received follow-up at 6 months either via clinic or telephone consultation. Due to the tertiary nature of referrals, follow-up barium swallow was not routinely performed post-FESD.

### Ethics Approval

The study protocol conforms to the ethical guidelines of the 1975 Declaration of Helsinki and was approved by the local NHS Research and Development department. Written consent was obtained from all study participants.

### Fluoroscopic Measurement Protocol for Zenker’s Diverticulum

**Fig. 1:**
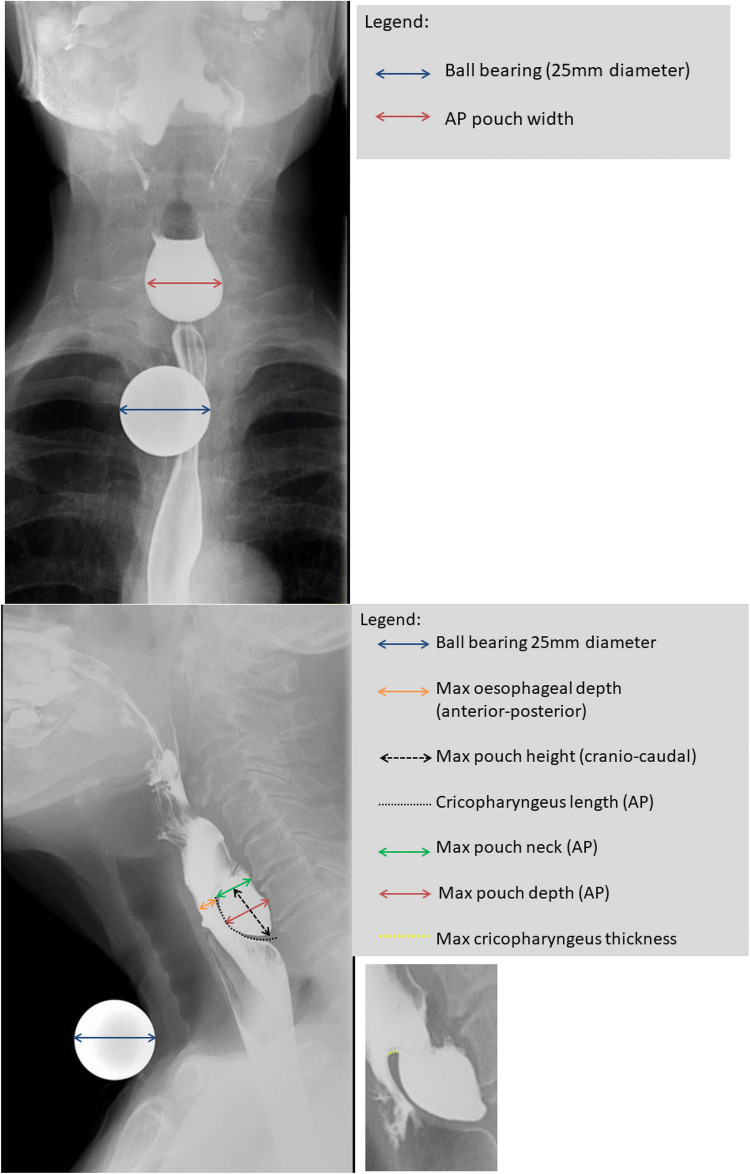
Protocol for the measurement of Zenker’s diverticulum on barium radiology. A 25 mm ball-bearing is used to calibrate distances. **a** Anteroposterior view, with measurement of pouch width; **b** lateral view, with measurement of maximum oesophageal depth, pouch height, cricopharyngeus length, pouch neck and pouch depth; **c** zoomed-in lateral view demonstrating maximum cricopharyngeal thickness

Fluoroscopic procedures were supervised by either accredited radiographers, radiology registrars or consultant radiologists in an upright position, and images were later reviewed by two specialist gastrointestinal radiologists (with 9 years and 22 years’ experience in Barium studies, respectively) to derive ZD measurements. The anteroposterior (AP) view showing the largest pouch dimensions was used to estimate the pouch width (Fig. 1a). The lateral view demonstrating the widest oesophageal luminal opening (Fig. 1b) was used as the image for maximum oesophageal depth and maximum pouch neck depth, both in a plane parallel to the vertebral endplates. Using the same image, the maximum craniocaudal pouch height was measured in a plane perpendicular to the vertebral end plates. The length of the cricopharyngeus muscle and maximum cricopharyngeus muscle thickness (inset) were also measured either on the lateral view or additional oblique views if these better demonstrated the relevant anatomy. The local examinations were performed on Siemen Luminous dRF, with a pulse rate of 7.5 frames per second and frame rate of 4 frames per second. Being a tertiary referral centre, externally acquired images were from a variety of sources with variable parameters.

To facilitate morphometric analysis for patients with ZD, a 25 mm ball-bearing (BB) was taped around the level of the sternal notch to avoid obscuring the ZD on the lateral view. For the AP view the BB was moved taped in a more lateral position at a rough estimation of the position of any ZD. This enabled an estimate of the ZD parameters by calibrating a known dimension in a similar plane to the ZD, from which other measurements could be extrapolated, and to minimise radiographic parallax error [8].

For patients who did not have the 25 mm BB for reference, e.g. examination performed externally, unexpected finding of ZD, or performed by a practitioner unfamiliar with the protocol of using a 25 mm BB, a surrogate landmark was used to estimate the above ZD measurements. The C4 vertebra was chosen as it is likely to be constant, is usually more readily identifiable than higher or lower cervical levels which may be obscured by overlying anatomy, and lies in the midline. The craniocaudal height of the C4 vertebra (mid-sagittal vertebral body height) was approximated to 14 mm [9], and the required measurements extrapolated accordingly. We have since abrogated use of the BB in favour of the simpler method of using the height of C4 to calibrate ZD measurements.

### Study Outcomes and Covariates

The primary outcome measured was therapeutic success, i.e. durable remission after single attempt at FESD. Symptom severity related to ZD was measured using two scoring systems: Dakkak score [10] and the Dysphagia, Regurgitation, Complications (DRC) scale (Supplementary Table 1) [5, 11]. Remission was defined as a Dakkak score of 0 and DRC score of ≤ 1. Patients met the primary outcome if they received only one attempt at FESD and achieved remission during their 6-month review. Data were collected on a standardised pro forma by a dedicated team member. Intraprocedural and post-procedural complications were recorded. Procedural times were calculated by subtracting the extubation time from the intubation time, which were recorded by the in-room anaesthetist. In addition to the protocolised ZD measurements, other covariates of interest included age, gender and previous surgical intervention.

### Statistical Analyses

All continuous variables were subjected to normality assessment using the Shapiro–Wilk test. Non-parametric variables were presented as medians with interquartile range (IQR), with univariable comparisons made using Mann–Whitney (2 groups) and Kruskal–Wallis tests (> 2 groups).

The inter-rater reliability (reproducibility) of ZD dimensions was evaluated. Measurements were independently undertaken by two radiologists for the first 10 cases as proof of concept. Intraclass correlation coefficients (ICCs) were calculated using average measures within a two-way mixed effects model, with consistency set as the model type. *P*-values were derived from corresponding F-tests. Reliability interpretation of ICC values were as follows: < 0.5: poor reliability, 0.5–0.75: moderate reliability, 0.75–0.9: good reliability, > 0.90: excellent reliability.

Multivariable linear regression analyses were conducted to identify predictors of ZD dimensions according to age, sex, symptoms (DRC score) and Zenker’s status (recurrence vs treatment naïve). A binary logistic regression model was also performed to identify factors associated with therapeutic success. Statistical analyses were performed in SPSS (v25, Arkmont, NY: IBM Corp), with *P* < 0.05 indicative of significance.

## Results

### Baseline Characteristics

Over the study period, a total of 67 patients (mean age 74.3; SD 11.4) were included for analysis. 41 (61.2%) were male and 30 (44.8%) had undergone previous surgical stapling. This cohort had significant co-morbidity, with 29 (43.3%) comprising American Society of Anaesthesiologist Grades III or IV. Baseline dysphagia severity scores, as measured using the DRC score, are presented in Supplementary Table 2. All but one patient reported dysphagic symptoms; this patient had a regurgitation score of 3 and complication score of 2.

### Reliability

Reliability coefficients (ICCs) are presented in Table 1. ICCs for all measurements exceeded 0.8, with the exception of the oesophageal depth dimension (ICC 0.018). ICCs for pouch width (0.981) and pouch depth (0.934) exceeded 0.9, indicating excellent reliability. Overall, the ICC for pouch width was highest, with a lower bound 95% CI of 0.925. Due to the poor reliability of oesophageal depth as a ZD dimension, this was removed from subsequent analyses.

**Table 1:** Interobserver reliability of Zenker’s diverticulum barium measurements as measured using intraclass correlation coefficients (ICCs)

Dimension	ICC	95% CI of ICC	*P*-value (*F*-test)
Oesophageal depth	0.018	− 2.95 to 0.756	0.489
CP length	0.891	0.562 to 0.973	0.001
CP thickness	0.822	0.284 to 0.956	0.008
Pouch neck	0.858	0.427 to 0.965	0.004
Pouch height	0.875	0.496 to 0.969	0.002
Pouch width	0.981	0.925 to 0.995	< 0.001
Pouch depth	0.934	0.734 to 0.984	< 0.001

### Fluoroscopic Dimensions

Fluoroscopic ZD dimensions were stratified by age, sex and according to whether the patient had undergone previous surgical intervention (Table 2). This found that male patients had significantly larger dimensions with regard to pouch height, pouch width and a trend towards significance for CP length and pouch depth. Patients with recurrent ZD who were selected for FESD had significantly larger pouch neck and pouch width dimensions compared to those without prior therapy. No significant differences in dimensions were found in patients aged > 75 vs. 75 or less.

**Table 2:** Baseline Zenker’s diverticulum measurements prior to flexible endoscopic septal division, with comparisons made according to gender, previous surgical intervention and age

Dimension (mm)	Median dimension in mm (IQR)								
	Gender			Previous surgical intervention			Age (years)		
	Male (*N* = 41)	Female (*N* = 26)	*P*-value	Yes (*N* = 30)	No (*N* = 37)	*P*-value	≤ 75 (*N* = 34)	> 75 (*N* = 33)	*P*-value
CP length	28.0 (17.9–31.9)	20.0 (13.6–24.1)	0.058	24.6 (15.9–33.3)	20.2 (16.9–31.1)	0.275	24.6 (16.8–31.9)	20.1 (16.9–28.7)	0.339
CP thickness	3.5 (2.3–4.3)	2.9 (1.8–4.0)	0.143	2.8 (1.6–4.0)	3.4 (2.3–4.2)	0.254	3.2 (1.7–4.1)	3.0 (2.0–4.1)	0.905
Pouch neck	10.7 (7.9–14.9)	10.3 (8.4–13.8)	0.891	12.4 (9.5–15.9)	9.2 (7.3–12.4)	0.010*	12.2 (9.0–18.6)	9.9 (7.6–12.2)	0.079
Pouch height	19.0 (11.7–31.8)	13.7 (10.4–17.3)	0.008*	17.9 (10.8–32.1)	14.0 (10.9–24.8)	0.075	17.9 (11.5–29.0)	13.9 (10.8–23.2)	0.930
Pouch width	28.0 (19.9–41.8)	20.0 (15.6–28.7)	0.006*	28.4 (20.7–41.7)	21.4 (17.3–29.2)	0.029*	28.4 (19.2–41.6)	22.6 (18.4–27.3)	0.339
Pouch depth	14.0 (10.7–24.4)	11.6 (9.0–17.4)	0.051	14.6 (10.90–22.6)	11.9 (7.4–16.2)	0.063	14.2 (10.0–23.1)	11.7 (9.7–15.6)	0.673

Bold values are statistically significant *P* value

*CP* cricopharyngeus

*P < 0.05

On multivariable linear regression analysis (Table 3) after accounting for age, sex, DRC and ZD status (recurrence vs naïve), male gender remained significantly associated with pouch height (*P* = 0.018), pouch width (*P* = 0.003) and pouch depth (*P* = 0.017), whereas previous intervention was associated with higher pouch height (*P* = 0.034).

**Table 3:** Multivariable linear regression analysis of factors associated with Zenker’s diverticulum dimensions

Dimension	Factor	Beta coefficient (mm)	95% Confidence interval	*P*-value
CP Length *B* = 9.5 mm *R*^2^ = 0.10	Age (per year)	− 0.03	− 0.32 to 0.26	0.833
	Sex (M vs F)	4.53	− 1.95 to 11.0	0.166
	DRC (per score)	0.88	− 0.87 to 2.63	0.316
	Recurrence vs naïve	4.67	− 1.87 to 11.2	0.158
CP thickness *B* = 3.8 mm *R*^2^ = 0.09	Age (per year)	− 0.01	− 0.04 to 0.03	0.645
	Sex (M vs F)	0.60	− 0.20 to 1.40	0.138
	DRC (per score)	− 0.07	− 0.30 to 0.17	0.564
	Recurrence vs Naïve	− 0.46	− 1.28 to 0.36	0.265
Pouch neck *B* = 9.4 mm *R*^2^ = 0.13	Age (per year)	− 0.08	− 2.1 to 0.06	0.248
	Sex (M vs F)	1.17	− 1.79 to 4.14	0.432
	DRC (per score)	0.63	− 0.17 to 1.43	0.119
	Recurrence vs naïve	2.29	− 0.71 to 5.29	0.131
Pouch height *B* = − 17.8 mm *R*^2^ = 0.16	Age (per year)	0.14	− 0.19 to 0.47	0.392
	Sex (M vs F)	9.44	1.66 to 17.21	0.018*
	DRC (per score)	0.88	− 1.13 to 2.89	0.384
	Recurrence vs Naïve	8.19	0.62 to 15.72	0.034*
Pouch width *B* = 15.2 mm *R*^2^ = 0.25	Age (per year)	− 0.216	− 0.52 to 0.09	0.161
	Sex (M vs F)	10.9	3.91 to 17.81	0.003*
	DRC (per score)	1.024	− 0.79 to 2.83	0.261
	Recurrence vs naïve	4.98	− 1.93 to 11.89	0.154
Pouch depth *B* = 0.6 mm *R*^2^ = 0.22	Age (per year)	− 0.049	− 0.23 to 0.13	0.595
	Sex (M vs F)	5.03	0.92 to 9.13	0.017*
	DRC (per score)	1.41	0.33 to 2.49	0.011*
	Recurrence vs naïve	2.851	− 1.27 to 6.97	0.171

Bold values are statistically significant *P* value

The Beta coefficient (Beta) denotes increases in dimension size from the constant (B) for each categorical factor, or for each unit increase for continuous variables

*M* male, *F* female, *DRC* Dysphagia, Regurgitation, Complications Scale

### Symptom Severity

ZD dimensions were also subjected to Spearman rank analyses against each subset of the DRC score in addition to the total score (Table 4). No statistically significant correlations were found between ZD measurements and individual subset scores for dysphagia (D) and complications (C), but was positive for regurgitation (R), which correlated with pouch depth (*ρ* = 0.330, *P* = 0.010) and pouch neck (*ρ* 0.267, *P* = 0.045) measurements. The composite DRC score correlated with pouch depth size (*ρ* 0.326, *P* = 0.011). This remained significant after multivariable analysis for age, sex and previous intervention (Table 3).

**Table 4:** Comparisons of Zenker’s diverticulum dimensions according to the primary outcome

Dimension	Dysphagia	Regurgitation	Complication	DRC score
CP length	− 0.012 *P* = 0.933	0.098 *P* = 0.478	0.029 *P* = 0.833	0.058 *P* = 0.676
CP thickness	0.025 *P* = 0.858	− 0.137 *P* = 0.332	− 0.133 *P* = 0.348	− 0.130 *P* = 0.359
Pouch neck	− 0.004 *P* = 0.978	**0.267** P = 0.045*	0.178 *P* = 0.186	0.242 *P* = 0.070
Pouch height	0.105 *P* = 0.396	0.078 *P* = 0.531	0.040 *P* = 0.751	0.094 *P* = 0.448
Pouch width	0.034 *P* = 0.803	0.135 *P* = 0.313	0.185 *P* = 0.164	0.153 *P* = 0.252
Pouch depth	0.078 *P* = 0.554	**0.330** P = 0.010*	0.165 *P* = 0.207	**0.326** P = 0.011*

Bold and italic values are statistically significant *P* value

*CP* cricopharyngeus

*P < 0.05

### Procedure Times

The median procedure time was 20 min (IQR 20.0–25.0). There were no significant correlation between any ZD dimension and procedure duration or according to whether the patient had undergone previous surgical intervention (*P* = 0.695). In the treatment naïve subgroup, pouch depth was the only dimension which showed a statistically significant correlation (*ρ* 0.358, *P* = 0.041) with procedure time.

### Correlations with Other ZD Dimensions

Bivariate correlation analyses between individual ZD dimensions were performed (Table 5). This revealed strong positive correlations between CP length and the pouch height (*ρ* 0.890, *P* < 0.001), pouch width (rho 0.719, *P* < 0.001) and pouch depth (*ρ* 0.719, *P* < 0.001), and a moderately positive correlation with the pouch neck (*P* = 0.546, *P* < 0.001), but not with CP thickness (*P* = 0.194). There were no significant correlations between CP thickness and other dimensions.

**Table 5:** Correlations between Zenker’s diverticulum dimensions. CP: cricopharyngeus

	CP length	CP thickness	Pouch neck	Pouch height	Pouch width	Pouch depth
CP length						
CP thickness	0.183 *P* = 0.194					
Pouch neck	**0.546** ***P < 0.001***	− 0.146 *P* = 0.301				
Pouch height	**0.890** ***P < 0.001***	0.056 *P* = 0.694	**0.423** ***P = 0.001***			
Pouch width	**0.719** ***P < 0.001***	− 0.034 *P* = 0.815	**0.618** ***P < 0.001***	**0.757** ***P < 0.001***		
Pouch depth	**0.786** ***P < 0.001***	− 0.062 *P* = 0.661	***P = 0.767*** ***P < 0.001***	**0.775** ***P < 0.001***	**0.791** ***P < 0.001***	

Bold and italic values are statistically significant *P* value

### Therapeutic Success

**Fig. 2:**
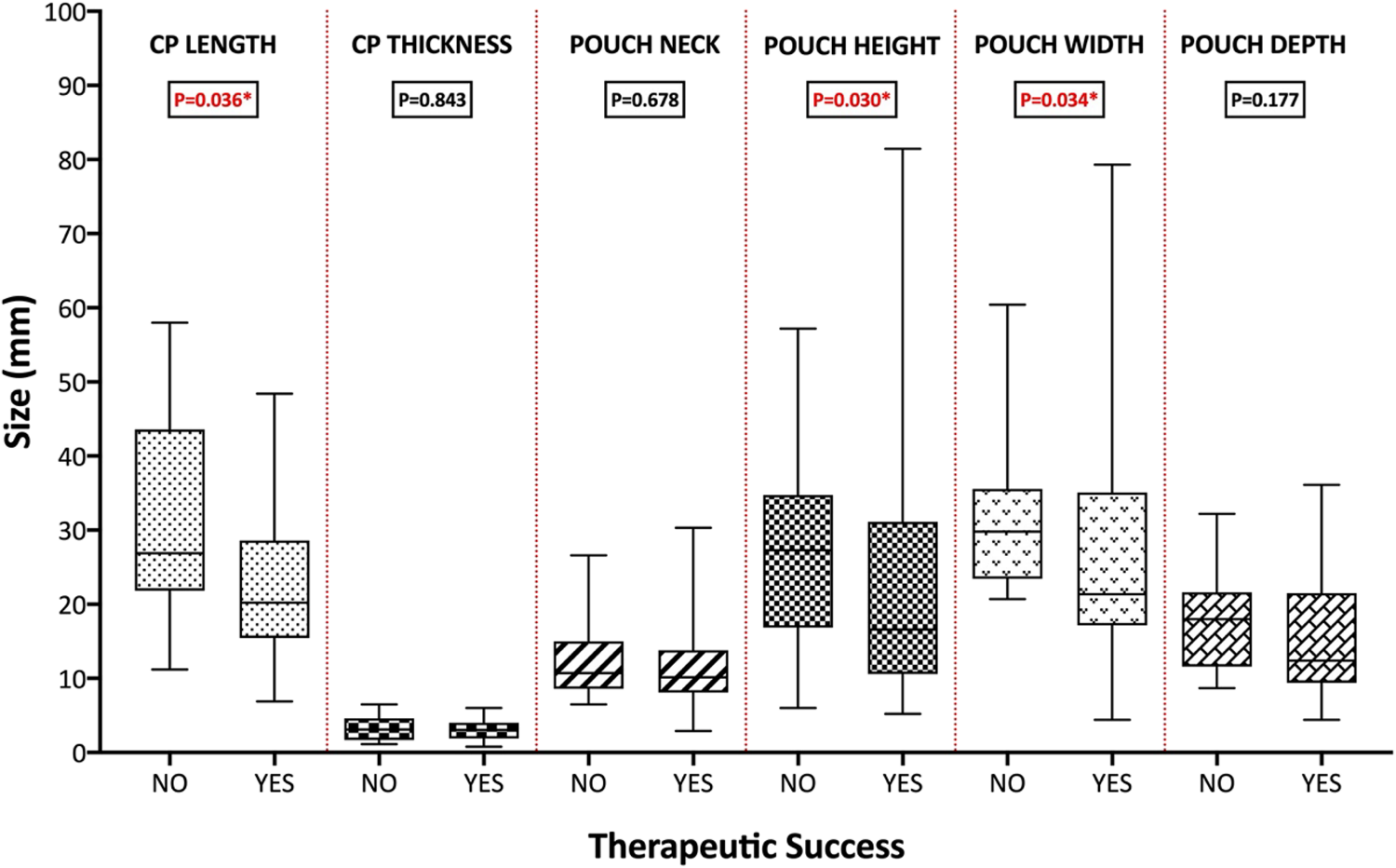
Comparisons of Zenker’s diverticulum dimensions according to the primary outcome. *CP* cricopharyngeus*.* *P < 0.05

The primary outcome of durable remission after first episode FESD was met in 64.2%. On univariable analysis (Fig. 2), therapeutic success was associated with shorter CP length (*P* = 0.036), pouch height (*P* = 0.030) and pouch width (*P* = 0.034). No significant differences were found on multivariable analyses after accounting for age, previous surgical intervention or gender.

## Discussion

Barium swallow is the primary radiological investigation for dysphagia [2]. The technique is dependent upon practitioner experience and local protocols, with variation in terms of radiographic projections and fluoroscopic capture rates. In order to standardise the assessment and reporting of ZD dimensions on barium fluoroscopy, we have developed a novel and purpose-specific protocol for quantifying ZD morphometrics. In this prospective single-centre study, we show that, by using this protocol, ZD measurements can be reproducible amongst GI radiologists, as evidenced by inter-rater reliability analyses (ICCs). Importantly, we demonstrate that specific dimensions correlated with symptom severity, procedure time and the outcome of durable therapeutic success. These results provide validity evidence in support of our ZD measurement protocol.

Although endoscopy is often first line in the evaluation of dysphagia, the hypopharynx represents a potential blind spot which may lead to a false negative diagnoses of ZD. It is also recognised that ZD can hinder endoscopic intubation of the oesophagus. Thus, there is a role for fluoroscopic assessment in patients with intubation failure [12], or in patients with oropharyngeal dysphagia for which a high index of suspicion for structural abnormality remains despite normal endoscopy. This may be particularly helpful in patients with regurgitation-predominant symptoms, as our analyses demonstrate a positive correlation between pouch dimensions and the regurgitation subset of the DRC symptom severity scale. Although designed to assess patients with endoscopically confirmed ZD for pre-FESD workup, our Barium protocol could also be adopted for use in the evaluation of oropharyngeal dysphagia.

In our experience, the anteroposterior and lateral views of the pharynx and upper oesophagus were most helpful for determining ZD morphometrics. An image acquisition rate of 3 or 4 frames per second (dependent on equipment manufacturer) was felt to be a pragmatic balance between minimising radiation exposure to as low as reasonably achievable (ALARA) and providing adequate temporal resolution for functional assessment to evaluate for pooling of contrast, regurgitation and aspiration, and mass effect on the adjacent oesophagus. Routine oblique views and spot radiographs of the ZD were not felt to be as useful with problematic quantification of the ZD measurements and inherently lacked real-time functional information, although some radiologists prefer oblique views as the ZD projects posterolaterally (and usually to the left). The externally referred images had a variety of pulse rates and frame rates, some being spot films, with inherent limitations of non-standardised image acquisition. The local operators comprised radiographers, radiology registrars an consultants and we presume a similar spread for external sites. Clearly given the radiation exposure we did not repeat examinations if the measurements could be derived from the referral source. We hope discussion of these factors will lead to an awareness of measurements used in ZD, particularly for patients who may be considered for FESD. Whilst we did use a ball-bearing for calibration, this was sometimes found to be cumbersome and could obscure assessment of the underlying anatomy, requiring repositioning during the examination. The utility of using a ball-bearing over calibrating from the ever-present posterior height of the C4 vertebral body was not separately investigated; however, given the inherently dynamic nature of ZD filling and opacification, theoretical discrepancies between the two methods were felt to be small and unlikely to have a significant impact on the ultimate procedural outcome. For these reasons, we suggest an alternative method for extrapolating ZD dimensions by using 14 mm as the height of the C4 vertebral body [9].

Correlations between ZD dimensions can provide insights on its pathophysiology. There was significant inter-correlation between the pouch height, width, depth and the CP length, but not CP thickness. Male patients were associated with significantly larger pouch height, depth and width, which complements the observation that ZD, is nearly twice as common in men [13]. The observation that patients with recurrent ZD had larger dimensions may be due to selection bias. It is possible that, in patients who have failed previous therapy, clinicians may have a higher threshold to refer for further endoscopic therapy.

Several limitations should be discussed. First, our study size was small (*N* = 67) which did not permit multivariable analyses of therapeutic efficacy. For the evaluation of reliability, only the first 10 cases were reviewed by two GI radiologists (blinded to the results) due to resource constraints, which may have influenced the precision of reliability estimates. Some cases were performed externally, which may affect the accuracy and consistency of ZD measurements. The success rate of 64.2% is lower than other series, and may be due to several factors. Our study outcome of therapeutic success was based on a stringent definition of durable remission at 6 months after index FESD. It should be noted that the definition of procedural success within the literature is heterogeneous. Some defined success as symptomatic improvement after three episodes of FESD, improvement of symptoms, or after 3 months of follow-up. Procedures were mainly performed in elderly patients (mean age of 74) with co-morbidity (43.4% had ASA grades of III or IV), of which a significant proportion (44.8%) of patients were patients with recurrent ZD who had previously failed endoscopic stapling. These factors may affect the generalisability of our data towards other patient populations. Finally, patients post-FESD may have a degree of bridging fibrosis, i.e. scarring, which may result in dysphagic symptoms but may not necessary have recurrent ZD. Due to the tertiary nature of referrals from throughout the UK, it was not feasible to repeat barium fluoroscopy in all patients post FESD. This may have been useful to confirm recurrence and to study pairwise comparisons of pre- and post-FESD dimensions. Indeed, there is little evidence that assessment of residual pouch size post-procedure predicts a successful outcome or future symptomatic recurrence [14, 15].

In summary, a measurement protocol for the assessment of ZD on barium radiology has been developed. These measurements are reproducible and correlate with symptom severity, procedure time and post-FESD outcomes, and may inform FESD planning and patient counselling of post-FESD outcomes. Further studies are required to inform whether volumetric analyses can be used in conjunction with Barium dimensions to benefit patient selection, procedural selection (e.g. FESD vs. submucosal tunnelling techniques) and ultimately, on patient outcomes. Further studies are required to inform whether volumetric analyses can be used in conjunction with Barium dimensions to benefit patient selection, procedural selection (e.g. FESD vs. submucosal tunnelling techniques) and ultimately, on patient outcomes.

### Clinical Applicability of Study Findings: What Gap in Evidence Does the Study Fill?

Despite its role in the diagnosis and functional assessment of ZD, there is no accepted protocol for the standardised reporting of ZD dimensions based on barium swallow. Accordingly, this study aimed to develop a protocol for the fluoroscopic measurement of ZD dimensions, assess for interobserver reliability, and to correlate these measurements with pre-treatment symptoms and post-treatment outcomes. This is the first study to describe measurement protocol for the assessment of ZD on barium radiology. These measurements are reproducible and correlate with symptom severity, procedure time and post-FESD outcomes, and may inform FESD planning and patient counselling of post-FESD outcomes. We hope this protocol can be tested in further studies before adoption for routine practice.

## Electronic supplementary material

Below is the link to the electronic supplementary material.

Supplementary file1 (DOCX 13 kb)

## Abbreviations

CP: Cricopharyngeus muscle
DRC Scale: Dysphagia, Regurgitation, Complications Scale
FESD: Flexible endoscopic septal division
ICC: Intraclass correlation coefficients
IQR: Interquartile range
ZD: Zenker’s diverticulum

## References

[1] Tao TY, Menias CO, Herman TE (2013). Easier to swallow: pictorial review of structural findings of the pharynx at barium pharyngography. Radiographics.

[2] Carucci LR, Turner MA (2015). Dysphagia revisited: common and unusual causes. Radiographics.

[3] Ishaq S, Sultan H, Siau K (2018). New and emerging techniques for endoscopic treatment of Zenker's diverticulum: state-of-the-art review. Dig Endosc.

[4] Ishaq S, Hassan C, Antonello A (2016). Flexible endoscopic treatment for Zenker's diverticulum: a systematic review and meta-analysis. Gastrointest Endosc.

[5] Antonello A, Ishaq S, Zanatta L (2016). The role of flexible endotherapy for the treatment of recurrent Zenker's diverticula after surgery and endoscopic stapling. Surg Endosc.

[6] Jaffer NM, Ng E, Au FW-F (2014). Fluoroscopic evaluation of oropharyngeal dysphagia: anatomic, technical, and common etiologic factors. Am J Roentgenol.

[7] Costamagna G, Iacopini F, Bizzotto A (2016). Prognostic variables for the clinical success of flexible endoscopic septotomy of Zenker's diverticulum. Gastrointest Endosc.

[8] Buckle CE, Udawatta V, Straus CM (2013). Now you see it, now you don’t: visual illusions in radiology. Radiographics.

[9] Gilad I, Nissan M (1985). Sagittal evaluation of elemental geometrical dimensions of human vertebrae. J Anat.

[10] Dakkak M, Bennett JR (1992). A new dysphagia score with objective validation. J Clin Gastroenterol.

[11] Battaglia G, Antonello A, Realdon S (2015). Flexible endoscopic treatment for Zenker's diverticulum with the SB knife. Preliminary results from a single-center experience. Dig Endosc.

[12] Siau K, Li J, Fisher NC (2017). Intubation failure during gastroscopy: incidence, predictors and follow-up findings. J Gastrointestin Liver Dis.

[13] Yeo JC, Mackenzie K (2010). Pharyngeal pouch surgery in north Glasgow: NICE (National Institute for Health and Clinical Excellence) practice or not?. J Laryngol Otol.

[14] Mantsopoulos K, Psychogios G, Karatzanis A (2014). Clinical relevance and prognostic value of radiographic findings in Zenker's diverticulum. Eur Arch Otorhinolaryngol.

[15] Ong CC, Elton PG, Mitchell D (1999). Pharyngeal pouch endoscopic stapling–are post-operative barium swallow radiographs of any value?. J Laryngol Otol.

