# Supplementary material for: Zenker’s Diverticulum: Can Protocolised Measurements with Barium SWALLOW Predict Severity and Treatment Outcomes? The “Zen-Rad” Study
Source: Dysphagia. 2020 Jun 19;36(3):393–401. doi: 10.1007/s00455-020-10148-5 (PMC8163680; doi:10.1007/s00455-020-10148-5)
Supplement: Supplementary file 1 — Supplementary file1 (DOCX 13 kb) [file 455_2020_10148_MOESM1_ESM.docx]

**Supplementary File**

| Score | **Dysphagia (D)** | **Regurgitation (R)** | **Complication (C)** |
| --- | --- | --- | --- |
| 0 | No dysphagia | No regurgitation | No complications |
| 1 | Dysphagia with normal/solid diet | More than once a week, less than once a day | Recurrent chest infections OR unintentional weight loss >5kg over last 3 months |
| 2 | Dysphagia with soft diet (semi solid) | At least once per day | Recurrent chest infections and unintentional weight loss |
| 3 | Dysphagia with fluids | Immediately following all meals/drinks |  |
| 4 | Difficulty swallowing saliva | Regurgitation resulting in choking or coughing |  |

**Supplementary Table 1**: Distribution of Zenker’s diverticulum symptom severity scores.

| Score | **Dysphagia (D)** | **Regurgitation (R)** | **Complication (C)** | **DRC Total** | |
| --- | --- | --- | --- | --- | --- |
| 0 | 1 (1.5%) | 14 (20.9%) | 36 (53.7%) | 2-3 | 15 (22.4%) |
| 1 | 16 (23.9%) | 10 (14.9%) | 24 (35.8%) | 4-5 | 28 (41.8%) |
| 2 | 22 (32.8%) | 7 (10.4%) | 7 (10.4%) | 6-7 | 18 (26.8%) |
| 3 | 23 (34.3%) | 16 (23.9%) | - | 8-9 | 4 (6.0%) |
| 4 | 5 (7.5%) | 20 (29.9%) | - | 10 | 2 (3.0%) |

**Supplementary Table 2**: Distribution of Zenker’s diverticulum symptom severity score.
